# Evaluation of Pediatric Patients Diagnosed with Cutaneous Leishmaniasis: A Single-Center Experience

**DOI:** 10.3390/tropicalmed11030071

**Published:** 2026-03-05

**Authors:** Melis Deniz, İsa An, Kerim Parlak, Hasan Tezer

**Affiliations:** 1 Department of Pediatric Infectious Diseases, Sanlıurfa Training and Research Hospital, Sanlıurfa 63250, Turkey; 2 Department of Pediatric Infectious Diseases, Basaksehir Cam and Sakura City Hospital, Istanbul 34480, Turkey; 3 Department of Dermatology and Venereology, Sanlıurfa Training and Research Hospital, Sanlıurfa 63250, Turkey; 4 Department of Microbiology, Sanlıurfa Training and Research Hospital, Sanlıurfa 63250, Turkey; 5 Department of Pediatric Infectious Diseases, Faculty of Medicine, Gazi University, Ankara 06560, Turkey

**Keywords:** leishmaniasis, cutaneous, children

## Abstract

Objective: We aimed to describe the clinical features and treatment outcomes of pediatric patients with leishmaniasis. Methods: This retrospective study included pediatric patients (>1 month–18 years) diagnosed with leishmaniasis at Şanlıurfa Training and Research Hospital between January 2022 and January 2024, identified from electronic medical records. Results: Among patients with cutaneous leishmaniasis, fifty pediatric patients were evaluated. Plaques (*n* = 34, 68%) and ulcerative lesions (*n* = 8, 16%) were the most common lesion types, with the face and neck being the most frequently affected sites (*n* = 34, 68%). The number of previously used antibiotics was statistically significantly higher in the multiple-lesion group compared to the single-lesion group (*p* = 0.022). Conclusions: Clinicians should consider cutaneous leishmaniasis in children with plaque or ulcerative skin lesions and a history of travel to an endemic area. Early detection and timely treatment can prevent long-term damage and cosmetic issues, leading to improved patient outcomes.

## 1. Introduction

Leishmaniasis is a tropical disease caused by a parasitic protozoan of the genus *Leishmania* [1]. Cutaneous leishmaniasis (CL) is prevalent in about 100 countries, with over 100,000 new cases reported annually in the Eastern Mediterranean region, likely indicating a much higher actual incidence [1,2]. *L. major*, *L. tropica*, and *L. infantum* are considered to be the causative agents of CL [3]. Leishmaniasis, transmitted by sandflies, is more common in areas with poor socioeconomic conditions and inadequate waste management, leading to increased sandfly breeding areas. Outbreaks of this parasitic disease can be caused by various factors such as malnutrition, climate change, and the movement of non-immune individuals into regions with high transmission rates [4]. Leishmaniasis has three different clinical forms, determined by a complex interplay between parasite-related factors such as virulence, species, and tropism and the host’s immune response [5]. The first form is localized cutaneous leishmaniasis (LCL), which can be identified by skin ulcers that can cause permanent scars and severe disability if left untreated. The second form is mucocutaneous leishmaniasis (MCL), which affects mucous membranes and connective tissues, and the third form, visceral leishmaniasis (VL), leads to symptoms such as fever and weight loss, and impacts internal organs such as the liver and spleen [1].

Cutaneous leishmaniasis is the most common form of the infection, causing painless sores at the site of sandfly bites. Boosting the immune system through cell-mediated immunity and anti-leishmanial treatment can aid recovery [5]. For accurate diagnosis, a skin biopsy can be performed on lesions, which permits testing for other diagnoses through histopathology and cultures [6]. Treatment for this disease varies depending on factors such as the type of parasite, the patient’s condition, and clinical characteristics. Considering these factors is essential in selecting the appropriate agent, dosage, and treatment duration [6]. There is a lack of sufficient evidence to support treatments for pediatric CL, highlighting the urgent need for further research in children. However, the inadequate treatment of CL can result in destructive lesions, chronic wounds, scarring, and superinfection. In addition, immunocompromised patients are at risk of spreading infections through the skin, mucous membranes, and internal organs [6]. When evaluating chronic skin lesions, it is essential to consider CL as a potential cause. Due to migration and travel, CL cases are increasingly being reported in non-endemic countries [1,7]. Clinicians in non-endemic regions may have limited experience in recognizing CL symptoms, leading to treatment delays. The early diagnosis and prompt treatment of CL can prevent long-term damage and cosmetic consequences and improve patient outcomes.

The aim of this study was to provide a detailed clinical description of pediatric CL patients and report the treatments administered and their associated outcomes at a hospital in Turkey over a two-year period. The objectives were to investigate the clinical features of CL in children, illustrate how inflammatory markers can aid in the diagnosis of the disease, and discuss the available treatment options and their effectiveness for CL in children. The goal was to enhance medical professionals’ understanding of pediatric CL, raise awareness about the disease, and contribute to the development of improved treatments.

## 2. Methods

### 2.1. Study Setting

The study was conducted at Şanlıurfa Training and Research Hospital, a tertiary care center located in the southeastern Anatolia region of Turkey. The study population reflects the heterogeneous demographic structure of the region, including both local residents of this endemic area and children from Syrian refugee families. Sanlıurfa Training and Research Hospital serves as a regional referral center for patients with complex leishmaniasis who require systemic treatment based on lesion characteristics and for patients with inadequate response to intralesional therapy.

### 2.2. Study Design and Population

The pediatric patients treated for leishmaniasis from one month to 18 years of age in Şanlıurfa Training and Research Hospital during the years January 2022 and January 2024 were retrospectively identified from the electronic medical record system using the International Classification of Diseases, 10th Revision (ICD-10) codes referring to a diagnosis of leishmaniasis (B55.0, B55.1, B55.2, B55.9).

### 2.3. Case Definitions

Patients with cutaneous leishmaniasis were only included in the study if the diagnosis was laboratory-confirmed. The diagnosis of cutaneous leishmaniasis was established based on compatible clinical findings together with the demonstration of *Leishmania* parasites by at least one of the following methods: direct microscopic examination of stained smears, histopathological examination, or culture. Upon retrospective review, it was determined that polymerase chain reaction (PCR) had not been performed during the diagnostic evaluation of the patients included in this study.

### 2.4. Data Collection and Analysis

All clinical data for the CL patients were collected using standardized forms. The following information was extracted from the patients’ medical records: sociodemographic characteristics (age, gender); clinical appearance of cutaneous lesions (such as morphology, type, area, and extent); complaints; laboratory findings (including white blood cell [WBC] count, absolute neutrophil count [ANC], absolute lymphocyte count [ALC], platelet count, and c-reactive protein [CRP] levels); number of antibiotics used previously; duration from the onset of symptoms to diagnosis; location of main lesion; presence of lymphangitis, adenopathy, and bacterial co-infection; diagnostic method; type of treatment provided; and treatment outcomes three months after treatment completion. All clinical and laboratory data were retrieved from the hospital’s electronic medical records and systematically reviewed by an experienced dermatologist to ensure diagnostic accuracy and data consistency.

Clinical follow-up was conducted every two weeks for the first two months and then monthly for the following six months. The success or failure of treatment was determined based on clinical assessment, considering that complete re-epithelialization may take up to three months.

Patients who showed clinical improvement (lesion recovery and re-epithelialization) after treatment were classified as cured. Patients who initially recovered after completing the full treatment but subsequently developed typical cutaneous lesions consistent with leishmaniasis within three months were considered to have relapsed.

The study was approved by the Harran University Faculty of Medicine Non-interventional Clinical Research Ethics Committee (Approval number: HRÜ/24.02.82).

### 2.5. Statistical Analysis

Statistical analyses were performed using IBM SPSS Statistics version 22.0 (IBM Corp., Armonk, NY, USA). The normality of data distribution was assessed using the Shapiro–Wilk test. Continuous variables were presented as means ± standard deviations (SDs) or medians with 25th–75th percentiles (interquartile ranges [IQRs]), while categorical variables were shown as numbers and percentages. Quantitative variables were compared between two groups using Student’s *t*-test for normally distributed data and the Mann–Whitney U test for non-normally distributed data. Categorical variables were analyzed using Fisher’s exact test. Correlations between non-normally distributed variables were evaluated using Spearman’s rho correlation coefficient. A *p*-value < 0.05 was considered statistically significant.

## 3. Results

Between January 2022 and January 2024, 53 pediatric patients were initially identified with a diagnosis of leishmaniasis. Of these, 50 patients with a confirmed and definitive diagnosis of cutaneous leishmaniasis (CL) were included in the study. One patient diagnosed with visceral leishmaniasis was excluded from the analysis due to the insufficient sample size. All included patients had localized cutaneous leishmaniasis (LCL), and none had mucocutaneous leishmaniasis (MCL).

The ages of the children ranged from 1 to 17 years, with a mean age of 6.89 ± 4.33 years. Of the participants, 54% were boys (*n* = 27) and 46% were girls (*n* = 23). Regarding age distribution, 34% were younger than 5 years, 52% were between 5 and 11 years, and 14% were aged 12 years or older.

The total WBC counts of the children ranged from 4.86 to 17.19 × 10^9^/L, with a median value of 8.71 × 10^9^/L (IQR: 6.80–11.04). ANC values ranged between 1.41 and 9.06 × 10^9^/L, with a median of 3.77 × 10^9^/L (IQR: 3.02–5.21). ALC values ranged from 1.00 to 10.94 × 10^9^/L, with a median of 3.23 × 10^9^/L (IQR: 2.43–4.73). Platelet counts ranged from 207 to 556 × 10^9^/L, with a median of 339 × 10^9^/L (IQR: 303.5–390.5). CRP levels ranged from 0.09 to 25 mg/L, with a median of 0.78 (IQR: 0.26–2.03). In most cases, CRP levels were low.

The duration from the onset of symptoms to diagnosis ranged from 2 to 10 months, with a median of 4 months (IQR: 3–5). All patients received a CL diagnosis more than one month after the onset of their symptoms. The number of previously used antibiotics ranged from 0 to 4, with a median of 2 (IQR: 0–3) (Table 1).

### 3.1. Clinical Presentation

A single lesion was identified in 62% of the children (*n* = 31), whereas 38% (*n* = 19) had multiple lesions. The lesion size was >5 cm in 14% of cases (*n* = 7) and ≤5 cm in 86% (*n* = 43).

**Figure 1:**
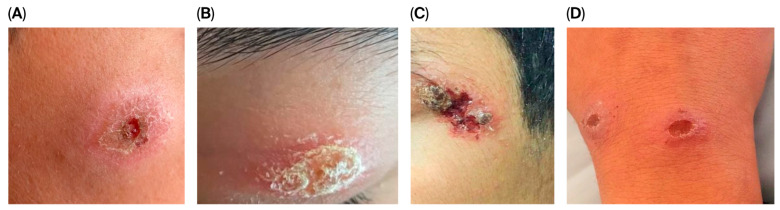
Skin lesions of four patients before treatment. (**A**) Erythematous crusted plaque with central ulceration. (**B**) Erythematous plaque with adherent crust. (**C**) Ulcerated crusted plaque with surrounding inflammatory erythema. (**D**) Ulcerative plaques with central depression.

Regarding lesion morphology, the most common type was plaque form, observed in 68% of patients (*n* = 34), followed by ulcerative lesions in 16% (*n* = 8). In addition, swollen/erythematous/indurated lesions were detected in 10% (*n* = 5), infiltrative type in 4% (*n* = 2), and nodular type in 2% (*n* = 1), as shown in Figure 1.

Complaint data were available for 26 of the 50 patients. Of the 26 patients, 57.7% (*n* = 15) reported pruritus as the main complaint, 30.7% (*n* = 8) had asymptomatic lesions, and 11.6% (*n* = 3) reported pain. Complaint information was missing for 24 patients due to incomplete documentation in the medical records.

A total of 84% of patients (*n* = 42) were diagnosed with CL through the direct microscopic examination of stained smears. Six patients (12%) were diagnosed through histopathological testing, while two patients (4%) were diagnosed through culture testing. Table 1 presents the clinical and demographic characteristics of the patients.

Adenopathy was present in 4% of the children (*n* = 2) and absent in 96% (*n* = 48). Bacterial coinfection was identified in 10% of cases (*n* = 5), whereas it was not detected in 90% (*n* = 45). Lymphangitis was not observed in any of the children (0%). Regarding localization, 68% (*n* = 34) of lesions were located in the head and neck region, 24% (*n* = 12) in the upper extremities, and 8% (*n* = 4) in the lower extremities (Table 2).

### 3.2. Treatments Administered

Out of the 50 patients, 49 received pentavalent antimony (SbV) [intramuscular (IM) meglumine antimoniate; dosage: 20 mg SbV/kg/day IM for 20 days] and one received intralesional (IL) antimony, as indicated in Table 1. One patient who was treated with IL antimony had a partial response and received intravenous liposomal amphotericin B as a second treatment, resulting in a cure. Among the 48 children whose treatment outcomes were evaluated, complete recovery was observed in 83.3%, partial response in 14.6%, and relapse in 2.1%.

### 3.3. Comparison of Patients with Single and Multiple Lesions

No statistically significant difference was observed between the single-lesion and multiple-lesion groups in terms of age (*p* = 0.990; *p* > 0.05) or total WBC values (*p* = 0.511; *p* > 0.05). No statistically significant difference was found between the single-lesion and multiple-lesion groups in terms of platelet values (*p* = 0.320; *p* > 0.05), and while CRP levels were higher in the multiple-lesion group, this difference did not reach statistical significance (*p* = 0.241; *p* > 0.05). No statistically significant difference was observed between the single-lesion and multiple-lesion groups in terms of symptom duration (*p* = 0.366; *p* > 0.05). The number of previously used antibiotics was statistically significantly higher in the multiple-lesion group compared to the single-lesion group (*p* = 0.022; *p* < 0.05). The proportion of patients whose largest lesion measured ≤5 cm was significantly lower in the single-lesion group (77.4%) compared to the multiple-lesion group (100%) (*p* = 0.035). A largest lesion size >5 cm was more frequently observed in the single-lesion group.

Adenopathy was not detected in the single-lesion group (0%), whereas it was observed in 10.5% (*n* = 2) of the multiple-lesion group; however, statistical comparison was not performed due to the low number of events. Bacterial coinfection was detected in 3.2% (*n* = 1) of the single-lesion group and in 21.1% (*n* = 4) of the multiple-lesion group; however, statistical analysis was not performed due to insufficient cell frequencies (Table 3).

In the single-lesion group, there was a moderate and statistically significant positive correlation between the duration of symptoms prior to admission and the number of previously administered antibiotics (r = 0.458; *p* = 0.010; *p* < 0.05). Within the multiple-lesion group, a statistically significant positive correlation was identified between the two variables (r = 0.832; *p* < 0.001) (Table 4).

**Figure 2:**
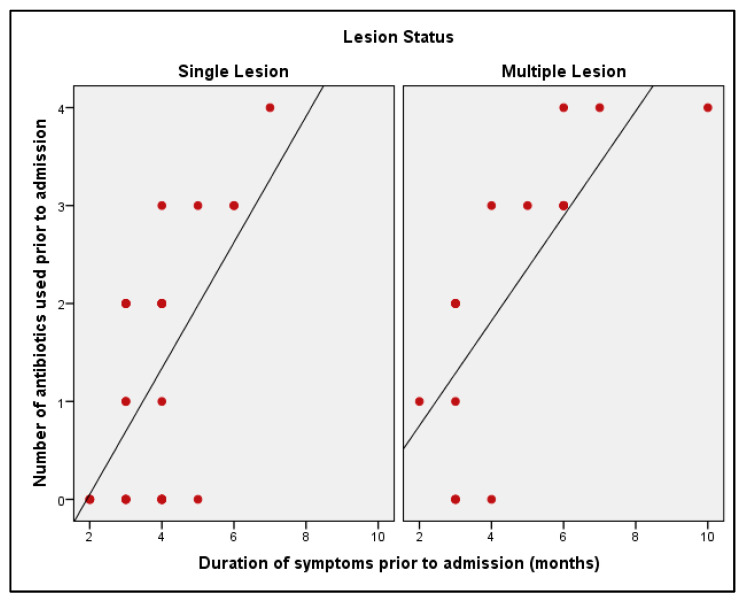
Relationship between symptom duration before admission and the number of previously used antibiotics in single- and multiple-lesion groups.

The results demonstrate that in both groups, the frequency of antibiotic use increases as the duration of symptoms lengthens. This association is especially apparent in the group with multiple lesions (Figure 2).

## 4. Discussion

According to our findings, the majority of patients presented after a prolonged duration of illness and extensive antibiotic use. All patients had LCL, with plaque and ulcerative lesions being the most common clinical manifestations. No mucosal leishmaniasis cases were observed during the study period at our hospital. The majority of the patients had a single lesion. Prolonged symptoms were linked to greater antibiotic use, especially among patients with multiple lesions. The diagnosis was primarily made through smears, and most received systemic therapy, mostly resulting in successful treatment.

Previous studies have revealed that CL cases have been reported in cities with hot and dry summers and cold winters with sub-zero temperatures. These climatic conditions are favorable for the growth and development of *Leishmania* and *Phlebotomus* sandflies [8]. Similar to previous studies, our study was conducted in Şanlıurfa, known for its hot and dry summer climate.

CL can manifest at any age. In a previous study, CL was most commonly seen in children aged 6–10 [9]. Previous studies have demonstrated that CL occurs more frequently among children between 7 and 12 years of age [10]. In our study, the highest incidence was observed among children aged 5–11 years, which is consistent with these findings. The observed distribution could be explained by higher levels of environmental contact and increased risk of sandfly exposure among children in this age group [10].

The sandflies that cause CL typically bite exposed areas of the body, such as the head, neck, arms, wrists, and hands, where the skin is thinner and more accessible [11]. Clothing is an effective barrier against sandflies, preventing their mouthparts from coming into contact with the skin [12].

The head and neck region was most affected by lesions in our study. Among children, lesions are most frequently reported to be localized on the cheek. This localization may reflect the increased susceptibility of the exposed head and neck areas, particularly the cheeks, to sandfly contact [13].

CL lesions start as small bumps at the inoculation site, developing into ulcers that can take 3 to 18 months to heal, depending on the type. In around 10% of cases, the condition becomes chronic and more severe [5]. Our hospital is a regional referral center, which may explain our patients’ high prevalence of chronic and severe lesions.

In our study, consistent with previous reports, most of the patients presented with a single lesion. Previous studies have reported higher rates of single-lesion presentations compared to our findings [14,15,16]. In our cohort, 62% of patients had a single lesion, which is lower than the proportions reported in previous studies, whereas the proportion of patients with multiple lesions (38%) was relatively higher. This difference may be related to regional patterns of the disease, delays in the diagnostic process, or socioeconomic features.

Our study shows that patients had undergone treatment with different types of antibiotics before being referred to our center. This delayed diagnosis may result in more complex conditions, necessitating systematic therapy for most of our patients.

Bacterial contamination has been reported in CL lesions in previous studies; in a cohort of 84 patients, *Staphylococcus aureus* (52.3%) was identified as the most commonly isolated pathogen [17]. This contamination can confuse clinicians, who may misdiagnose the condition as only a bacterial skin infection. We found a clear positive association between the duration of symptoms before admission and the number of antibiotics used in both groups. This link was much stronger in patients with multiple lesions. The findings indicate that persistent symptoms increase the likelihood of repeated antibiotic prescriptions prior to an accurate diagnosis, particularly among patients with multiple lesions. These findings underscore the importance of recognizing CL early to avoid unnecessary antibiotic use.

In our study, CL presented with a wide range of clinical manifestations. The diverse clinical spectrum of CL is believed to reflect the interplay between parasite virulence characteristics and the host’s immune response [10,18].

*Leishmania* infection elicits a cell-mediated immune response, and its outcome depends on the host’s Th1/Th2 response. Studies show that neutrophils migrate to the site of infection within 30 min and initiate phagocytosis [19]. There was no statistically significant difference between CRP levels, thrombocyte count, or WBC count in patients with single and multiple lesions. Similarly to our study findings, the literature indicates that WBC and CRP levels remain normal even in patients with disseminated CL [20]. High WBC and CRP levels may indicate bacterial infections, but they can be normal in patients with CL.

Cutaneous leishmaniasis may rarely progress to mucocutaneous disease, which can result in potentially severe outcomes. Infection with *Leishmania braziliensis* (*L. braziliensis*) is particularly associated with mucosal leishmaniasis (ML) [21]. ML is thought to develop through hematogenous or lymphatic dissemination from the primary cutaneous lesion, and although rare, fatal cases have been reported [22]. In our study, no patient developed mucosal involvement. An increased risk of ML has been documented in the southern Amazon region, including parts of Peru and Brazil [21]. An elevated risk of progression to ML has been linked to infection with *L. braziliensis*, as well as to larger or multiple lesions, longer lesion persistence, and head and neck involvement [23]. Therefore, ML should be considered in patients from high-risk regions who present with these features, and early diagnosis is essential to prevent destructive complications [21,23].

Clinicians should consider CL in patients with chronic skin lesions and a history of exposure in endemic areas. Confirmation of the diagnosis requires skin samples demonstrating the presence of *Leishmania* parasites [2]. In our study, most patients were diagnosed via visualization of the parasites in the smear.

For the effective treatment of CL with simple lesions, local treatment is strongly preferred, such as cryotherapy or intralesional injections of pentavalent antimonial drugs. Complex CL cases, as defined by the Infectious Diseases Society of America (IDSA), involve subcutaneous nodules, facial lesions, large regional adenopathy, multiple substantial-sized skin lesions, lesions that cannot be treated locally, and the clinical failure of local therapy [6]. The IDSA recommends systemic treatment for complex CL patients [6]. This may explain our study’s high systemic treatment rate because almost all of our patients’ lesions were consistent with this complex lesion definition. Systemic treatment options include conventional amphotericin B deoxycholate, lipid formulations of amphotericin B, pentavalent antimonial drugs (sodium stibogluconate and meglumine antimoniate), and pentamidine [6]. The WHO recommends systemic antimony for severe or complex lesions [4]. Antimonials are often preferred for treating CL due to their success and lower toxicity than amphotericin B deoxycholate [6]. In our study, most patients were treated with meglumine antimoniate, resulting in a high treatment success rate. Studies have shown that pentavalent antimonial compounds have resulted in complete cure rates ranging from 84% to 91% [17,24]. Studies on the treatment of pediatric patients with CL using liposomal amphotericin B have demonstrated varying results. One study showed positive outcomes, while the others observed complete clinical improvement ranging from 31% to 46% [25,26,27]. We successfully treated one patient with liposomal amphotericin B, leading to a complete cure.

This study provides valuable clinical data on pediatric cutaneous leishmaniasis cases managed in our center. However, the limitations should be acknowledged. Due to its retrospective design, PCR and species identification were not performed. Detailed migration status was not systematically documented in the medical records, limiting an assessment of the geographical distribution of cases.

## 5. Conclusions

This study provides crucial information about the clinical presentation and management of pediatric patients with CL, which is essential for public health. The current study findings revealed that all patients had experienced symptoms lasting more than one month, indicating a prolonged duration of illness. Additionally, most patients had previously used antibiotics as a form of treatment. Further prospective studies are essential for obtaining more precise data on treating pediatric patients with CL. Clinicians should consider CL in the differential diagnosis of children with plaque or ulcerative skin lesions and who have a previous history of traveling to an area where leishmaniasis is prevalent. It is essential to note that acute-phase reactants may remain within normal limits, even in cases where children with CL present with multiple lesions. Prospective studies incorporating molecular typing and comprehensive epidemiological data are warranted to better define species-specific clinical characteristics.

## Figures and Tables

**Table 1:** The clinical and demographic features of patients diagnosed with cutaneous leishmaniasis.

	Min–Max	Mean ± SD
Age (years)	1–17	6.89 ± 4.33
	**Min–Max**	**Median (IQR)**
Laboratory Findings		
Total WBC (×10^9^/L)	4.86–17.19	8.71 (6.80–11.04)
ANC (×10^9^/L)	1.41–9.06	3.77 (3.02–5.21)
ALC (×10^9^/L)	1.00–10.94	3.23 (2.43–4.73)
Plt (×10^9^/L)	207–556	339 (303.5–390.5)
CRP (mg/L)	0.09–25	0.78 (0.26–2.03)
Duration of symptoms (months)	2–10	4 (3–5)
Number of previously used antibiotics	0–4	2 (0–3)
	***n***	**%**
Gender		
Male	27	54
Female	23	46
Age Groups		
<5 years	17	34
5–11 years	26	52
≥12 years	7	14
Treatment Received		
IM meglumine antimoniate	49	98
IL meglumine antimoniate	1	2
Initial Treatment Outcomes (*n* = 48) ^¥^		
Partial response	7	14.6
Complete recovery	40	83.3
Relapse	1	2.1
Diagnostic Methods		
Smear	42	84
Histopathology	6	12
Culture	2	4

WBC: white blood cell count, ANC: absolute neutrophil count, ALC: absolute lymphocyte count, Plt: platelet, CRP: c-reactive protein, IM: intramuscular, IL: intralesional, SD: standard deviation; IQR: interquartile range (25–75%). ^¥^: Two patients’ data missing.

**Table 2:** The exact type and distribution of cutaneous leishmaniasis lesions.

	*n*	%
Number of lesions		
Single	31	62
Multiple	19	38
Largest lesion size		
>5 cm	7	14
≤5 cm	43	86
Lesion morphology		
Ulcerative	8	16
Plaque	34	68
Nodular	1	2
Swollen/Erythematous/Indurated	5	10
Infiltrative	2	4
Lymphangitis		
Present	-	-
Absent	50	100
Adenopathy		
Present	2	4
Absent	48	96
Bacterial coinfection		
Present	5	10
Absent	45	90
Lesion localization		
Head/Neck	34	68
Upper extremity	12	24
Lower extremity	4	8

**Table 3:** Comparison of clinical and laboratory features of patients with single and multiple lesions.

	Single Lesion	Multiple Lesions	*p*
	Mean ± SD	Mean ± SD	
Age (years)	6.97 ± 4.38	6.77 ± 4.37	^1^ 0.990
	**Median (IQR)**	**Median (IQR)**	
Laboratory findings			
Total WBC (×10^9^/L)	8.77 (7.28–11.03)	8.54 (6.10–11.05)	^2^ 0.511
Plt (×10^9^/L)	335.5 (301.5–364.3)	356 (306–408)	^2^ 0.320
CRP (mg/L)	0.47 (0.25–1.69)	0.87 (0.38–2.17)	^2^ 0.241
Duration of symptoms (months)	4 (3–4)	4 (3–6)	^2^ 0.366
Number of previously used antibiotics	1 (0–2)	2 (1–3)	^2^ 0.022 *
	***n*** ** (%)**	***n*** ** (%)**	
Largest lesion size			
>5 cm	7 (22.6%)	0 (0%)	^3^ 0.035 *
≤5 cm	24 (77.4%)	19 (100%)	
Adenopathy	0 (0%)	2 (10.5%)	–
Bacterial coinfection	1 (3.2%)	4 (21.1%)	–

^1^ Student’s *t* test. ^2^ Mann–Whitney U test. ^3^ Fisher’s exact test. Note: “–“ indicates that a statistical comparison could not be performed due to insufficient case numbers. WBC: white blood cell, Plt: platelet, CRP: c-reactive protein, SD: standard deviation; IQR: interquartile range (25–75%). * *p* < 0.05 considered statistically significant.

**Table 4:** Association between symptom duration and number of previously used antibiotics in patients with single and multiple lesions.

	Duration of Symptoms Prior to Admission (Months) - Number of Antibiotics Used Prior to Admission	
	r	*p*
Single Lesion	0.458	0.010 *
Multiple Lesions	0.832	<0.001 *

Spearman’s rho correlation analysis; * *p* < 0.05 considered statistically significant.

## Data Availability

The datasets generated and/or analyzed during the current study are not publicly available to ensure the privacy of the study participants, but are available from the corresponding author on reasonable request.

## References

[1] De Vries H.J.C., Schallig H.D. (2022). Cutaneous Leishmaniasis: A 2022 Updated Narrative Review into Diagnosis and Management Developments. Am. J. Clin. Dermatol..

[2] Van Kesteren L., Maniewski U., Bottieau E., Cnops L., Huits R. (2020). Cutaneous Leishmaniasis in Syrian Refugee Children: A Case Series. Pediatr. Infect. Dis. J..

[3] Solomon M., Astman N., Warshavsky K., Barzilai A., Meningher T., Avni D., Schwartz E. (2023). Cutaneous Leishmaniasis Caused by Leishmania infantum, Israel, 2018–2021. Emerg. Infect. Dis..

[4] WHO Technical Report Series 949. “Control of the Leishmaniases.” 2010. https://www.who.int/publications/i/item/WHO-TRS-949.

[5] Gurel M.S., Tekin B., Uzun S. (2020). Cutaneous leishmaniasis: A great imitator. Clin. Dermatol..

[6] Aronson N., Herwaldt B.L., Libman M., Pearson R., Lopez-Velez R., Weina P., Carvalho E.M., Ephros M., Jeronimo S., Magill A. (2016). Diagnosis and Treatment of Leishmaniasis: Clinical Practice Guidelines by the Infectious Diseases Society of America (IDSA) and the American Society of Tropical Medicine and Hygiene (ASTMH). Clin. Infect. Dis..

[7] Hill N.M., Irwin A.M., Graham N.M., Leung C.M., Francis J.R.M., Wall N.M., Nourse C. (2021). Treatment of Cutaneous Leishmaniasis in a Nonendemic Country: A Case Series of Children in Australia. Pediatr. Infect. Dis. J..

[8] Rather S., Wani M., Shah F.Y., Bashir S., Yaseen A., Giri F.A., Sharma R., Zeerak S., Jabeen Y., Hassan I. (2021). Clinical and epidemiological study of cutaneous leishmaniasis in two tertiary care hospitals of Jammu and Kashmir: An emerging disease in North India. Int. J. Infect. Dis..

[9] Sharma R.C., Mahajan V.K., Sharma N.L., Sharma A. (2003). A new focus of cutaneous leishmaniasis in Himachal Pradesh (India). Indian J. Dermatol. Venereol. Leprol..

[10] Rather S., Yaseen A., Shah F.Y., Wani M., Krishan K., Zirak S., Sharma R., Hassan I., Dogra D. (2022). Pediatric Cutaneous Leishmaniasis: A Clinico-Epidemiological Study from North India. Indian Dermatol. Online J..

[11] Uzun S., Gürel M.S., Durdu M., Akyol M., Karaman B.F., Aksoy M., Aytekin S., Borlu M., Doğan E.İ., Doğramacı Ç.A. (2018). Clinical practice guidelines for the diagnosis and treatment of cutaneous leishmaniasis in Turkey. Int. J. Dermatol..

[12] Ephros M., Aronson N.E., Long S.S., Prober C.G., Fischer M. (2018). *Leishmania* Species (Leishmaniasis). Principles and Practice of Pediatric Infectious Diseases.

[13] Kaya O.M., Serarslan G., Dirican E. (2020). Evaluation of clinical and demographic characteristics of Turkish and Syrian pediatric cutaneous leishmaniasis patients from Hatay, Turkey after the Syrian civil war. Adv. Dermatol. Allergol..

[14] Bari A.U. (2008). Childhood cutaneous leishmaniasis. J. Clin. Diagn. Res..

[15] Agrawal S., Khandelwal K., Bumb R.A., Oghumu S., Salotra P., Satoskar A.R. (2014). Short report: Pediatric cutaneous leishmaniasis in an endemic region in India. Am. J. Trop. Med. Hyg..

[16] Sharifi I., Fekri A.R., Aflatonian M.R., Nadim A., Nikian Y., Kamesipour A. (1998). Cutaneous leishmaniasis in primary school children in the south-eastern Iranian city of Bam, 1994–1995. Bull. World Health Organ..

[17] Layegh P., Ghazvini K., Moghiman T., Hadian F., Zabolinejad N., Pezeshkpour F. (2015). Bacterial Contamination in Cutaneous Leishmaniasis: Its Effect on the Lesions’ Healing Course. Indian J. Dermatol..

[18] Akilov O.E., Khachemoune A., Hasan T. (2007). Clinical manifestations and classification of Old World cutaneous leishmaniasis. Int. J. Dermatol..

[19] Ameen M. (2010). Cutaneous leishmaniasis: Advances in disease pathogenesis, diagnostics and therapeutics. Clin. Exp. Dermatol..

[20] Cataño J.C., Pinzón M.A. (2019). Disseminated Cutaneous Leishmaniasis in a Patient Infected by Leishmania panamensis. Am. J. Trop. Med. Hyg..

[21] Solomon M., Sahar N., Pavlotzky F., Barzilai A., Jaffe C.L., Nasereddin A., Schwartz E. (2019). Mucosal Leishmaniasis in Travelers with Leishmania braziliensis Complex Returning to Israel. Emerg. Infect. Dis..

[22] Jaimes J.R. (2022). Severe mucosal leishmaniasis with torpid and fatal evolution. Clin. Case Rep..

[23] Machado-Coelho G.L., Caiaffa W.T., Genaro O., Magalhães P.A., Mayrink W. (2005). Risk factors for mucosal manifestation of American cutaneous leishmaniasis. Trans. R. Soc. Trop. Med. Hyg..

[24] Heleine M., Elenga N., Njuieyon F., Martin E., Piat C., Pansart C., Couppie P., Hernandez M., Demar M., Blaizot R. (2023). Using pentamidine to treat cutaneous leishmaniasis in children: A 10-year study in French Guiana. Clin. Exp. Dermatol..

[25] Erat T., An I. (2022). Treatment of pediatric cutaneous leishmaniasis with liposomal amphotericin B. Dermatol. Ther..

[26] Guery R., Henry B., Martin-Blondel G., Rouzaud C., Cordoliani F., Harms G., Gangneux J.-P., Foulet F., Bourrat E., Baccard M. (2017). Liposomal amphotericin B in travelers with cutaneous and muco-cutaneous leishmaniasis: Not a panacea. PLoS Neglected Trop. Dis..

[27] Ubals M., Bosch-Nicolau P., Sánchez-Montalvá A., Salvador F., Aparicio-Español G., Sulleiro E., Silgado A., Soriano-Arandes A., Espiau M., Ferrer B. (2021). Treatment of Complex Cutaneous Leishmaniasis with Liposomal Amphotericin B. Pathogens.

